# Biomimetic Full-Thickness Artificial Skin Using Stromal Vascular Fraction Cells and Autologous Keratinocytes in a Single Scaffold for Wound Healing

**DOI:** 10.3390/bioengineering12070736

**Published:** 2025-07-05

**Authors:** Jung Huh, Seong-Ho Jeong, Eun-Sang Dhong, Seung-Kyu Han, Kyung-Chul Moon

**Affiliations:** 1 Korea University College of Medicine, Seoul 02841, Republic of Korea; 2 Department of Plastic Surgery, Korea University College of Medicine, Seoul 02841, Republic of Korea

**Keywords:** artificial skin, keratinocyte, stromal vascular fraction, wound healing

## Abstract

We developed biomimetic full-thickness artificial skin using stromal vascular fraction (SVF) cells and autologous keratinocytes for the dermal and epidermal layers of skin, respectively. Full-thickness artificial skin scaffolds were fabricated using 4% porcine collagen and/or elastin in a low-temperature three-dimensional printer. Two types of scaffolds with collagen-to-elastin ratios of 100:0 and 100:4 were printed and compared. The scaffolds were analyzed for collagenase degradation, tensile strength, and structural features using scanning electron microscopy. By 24 h, the collagen-only scaffolds showed gradual degradation, and the collagen-elastin scaffolds retained the highest structural integrity but were not degraded. In the tensile strength tests, the collagen-only scaffolds exhibited a tensile strength of 2.2 N, while the collagen-elastin scaffolds showed a tensile strength of 4.2 N. Cell viability tests for keratinocytes displayed an initial viability of 89.32 ± 3.01% on day 1, which gradually increased to 97.22 ± 4.99% by day 7. Similarly, SVF cells exhibited a viability of 93.68 ± 1.82% on day 1, which slightly improved to 97.12 ± 1.64% on day 7. This study presents a novel strategy for full-thickness artificial skin development, combining SVF and keratinocytes with an optimized single collagen scaffold and a gradient pore-density structure.

## 1. Introduction

Wound healing is a complex biological process through which the skin restores its integrity following injury. The skin consists of the following two primary layers: the epidermis, which forms the outermost barrier, and the dermis, located beneath it. In superficial or partial-thickness wounds, where damage is confined to the epidermal or the upper dermal layer, regeneration occurs primarily in the epidermis, leading to rapid healing with minimal scar formation. In contrast, deep wounds that extend beyond the mid-dermal layer, particularly those involving skin avulsion or the exposure of subcutaneous fat, are more prone to complications such as infection, and typically result in permanent scarring, even after complete healing [1].

Selecting an appropriate wound healing strategy based on the wound’s condition is essential for achieving optimal healing outcomes. Proper selection can reduce the risk of complications, accelerate the healing process, and minimize scar formation after complete wound closure. Various approaches have been employed in wound management, including healing by primary intention, secondary intention, tertiary intention, and the use of skin grafts and flaps [2,3]. Primary, secondary, and tertiary intentions are suitable for small wounds, but larger wounds that cannot be healed by these methods may require skin grafting or flap reconstruction. However, donor site morbidity may occur in skin grafting or flap reconstruction, and the microsurgical procedures involved may pose a physical burden to elderly patients [1].

Advancements in wound healing technology have led to the development of various artificial dermal substitutes [4,5,6,7,8]. However, these substitutes often result in noticeable and undesirable scarring [9,10]. In particular, wound contraction, a critical factor in minimizing scar formation after complete healing, remains a common challenge. While artificial dermis can be used alone for wound coverage, the absence of cell components frequently leads to delayed healing and abnormal scar formation, including scar contractures. Recent progress in wound healing technology has facilitated the adoption of cell-based therapies as an alternative to conventional treatments [11,12,13,14,15]. Cell therapy has the potential to enhance wound healing without requiring major surgical interventions or causing donor-site morbidity. This approach is applicable to both acute and chronic wounds, offering a promising strategy for improved tissue regeneration [1].

Both autologous and allogeneic cells can be used clinically in cell therapy for wound healing. However, the use of tissue-engineered skin containing allogeneic cells is limited due to regulatory challenges, such as the need for Food and Drug Administration approval and the completion of required clinical trials. Autologous cell-based therapy accelerates the wound healing process by reducing the time required for host cells to infiltrate wound tissue and by promoting the early synthesis of new skin [16,17]. Previous studies have shown that cell-only treatments can speed wound healing; however, they do not significantly improve wound contraction [18,19]. As a result, cells are often combined with artificial dermis to create a tissue-engineered dermal substitute that optimizes the healing process. Cell-seeded artificial dermal grafts minimize wound contraction while ensuring proper healing, making them particularly effective for treating skin and soft tissue defects [11]. Currently, the only tissue-engineered skin available for clinical use is one that involves seeding a single cell type onto an artificial dermis.

Therefore, the purpose of this in vitro study was to examine the possibility of developing tissue-engineered full-thickness artificial skin for clinical use to accelerate wound healing. We developed biomimetic full-thickness artificial skin using stromal vascular fraction (SVF) cells and autologous keratinocytes for the dermal and epidermal layers of skin, respectively. Our objectives included establishing a rapid fabrication protocol that maintains cell functionality, achieving structural organization that closely mimics native skin architecture, and evaluating the construct’s mechanical stability and biocompatibility. By combining the structural support necessary for both cell types within a single scaffold, our approach aims to simplify the manufacturing process while maintaining the essential biological functions of artificial skin. The development of this gradient scaffold system could provide a more practical and cost-effective solution for wound healing applications, potentially bridging the gap between laboratory innovations and clinical requirements in tissue engineering. This approach may represent a significant advancement over existing substitutes by combining the regenerative potential of SVF cells with the epidermal function of keratinocytes in a structurally biomimetic construct for healing full-thickness skin and soft tissue defects.

## 2. Materials and Methods

### 2.1. Preparation of Stromal Vascular Fraction Cells for the Dermal Layer

This study protocol was approved by the Institutional Review Board, and written informed consent was obtained from the patient prior to surgery. This study was conducted in full accordance with the principles of the Declaration of Helsinki.

Abdominal adipose tissue was collected from a 59-year-old female patient with no underlying health conditions who was undergoing liposuction under local anesthesia. A small stab incision was made in the umbilical region, followed by the infiltration of local anesthetic solution using a blunt Lamis infiltration cannula. The anesthetic solution comprised 2% lidocaine with epinephrine at a concentration of 1:200,000. The volume of infiltrated solution was typically twice the predicted amount of adipose tissue to be harvested.

Adipose tissue was aspirated using a 50 mL syringe attached to a cannula with a 3 mm inner diameter, designed to preserve the integrity of the adipose tissue parcels. The plunger of the syringe was gently retracted during aspiration to minimize excessive negative pressure and prevent adipose tissue rupture. Following tissue collection, the incision site was closed using interrupted nylon sutures. The average volume of harvested adipose tissue was 50 mL.

The collected adipose tissue was processed for autologous SVF cell isolation using an automated isolation system (Cellunit, CGBio Inc., Seoul, Republic of Korea). This system included a device and sterile disposable cartridges designed for tissue digestion, washing, and waste collection. Briefly, the harvested samples underwent enzymatic digestion with 0.1% collagenase (SERVA Electrophoresis GmbH, Heidelberg, Germany) within a processing chamber. The digested tissue was washed with saline to remove residual collagenase and then centrifuged to isolate SVF cells, which were obtained within 50 min. The total cell number and the number of nucleated SVF cells were determined using an automated cell counter (Luna-FL^TM^; Logos Biosystems, Anyang-si, Republic of Korea). The total number of SVF cells was 3.5 × 10^7^ cells at a final concentration of 9.7 × 10^5^ cells/mL.

### 2.2. Preparation of Keratinocytes for the Epidermal Layer

Keratinocytes were obtained from a skin biopsy and cultured in Dulbecco’s Modified Eagle Medium (DMEM, Gibco, Waltham, MA, USA) supplemented with 10% fetal bovine serum (FBS, Gibco) and 1% penicillin-streptomycin (Gibco). The cultures were maintained at 37 °C in a humidified atmosphere with 5% CO_2_. When the adherent monolayer reached 70–80% confluence, typically within 1 to 3 weeks, the cells were detached using TrypLE Express (Gibco) containing 0.25% trypsin. The detached cells were then washed, resuspended in DMEM supplemented with 10% FBS, and sub-cultured every 2 to 3 days. The cells were incubated for 24 h.

### 2.3. Scaffolds for Full-Thickness Artificial Skin

The thickness of the fibers and the spacing between each fiber were set at 0.2 mm. Scaffold printing was achieved by dispensing the material through a syringe onto a cooling plate. Although the full-thickness artificial skin scaffold can be printed up to 5 mm, this study produced a scaffold with a thickness of 2 mm, consisting of 10 stacked layers. The printed samples were subsequently freeze-dried and cross-linked to enhance their shape and functional performance.

**Figure 1:**
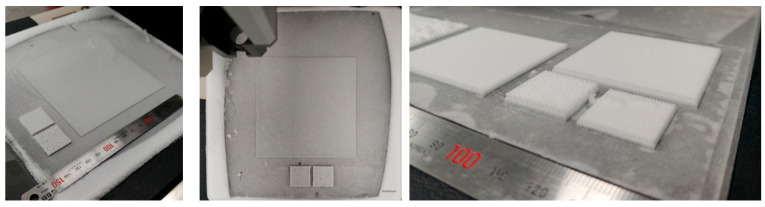
Fabrication of scaffolds for full-thickness artificial skin using a low-temperature three-dimensional printer. Scaffolds for full-thickness artificial skin were fabricated using 4% porcine collagen and/or elastin in a low-temperature three-dimensional printer.

The scaffolds for full-thickness artificial skin were fabricated using 4% porcine collagen and/or elastin in a low-temperature three-dimensional printer. Scaffolds with collagen-to-elastin ratios of 100:0 and 100:4 were printed. A three-dimensional printer was used to create a bi-layered structure, which was chemically cross-linked for mechanical stability and freeze-dried to develop a multiporous architecture for cell fixation (Figure 1).

**Figure 2:**
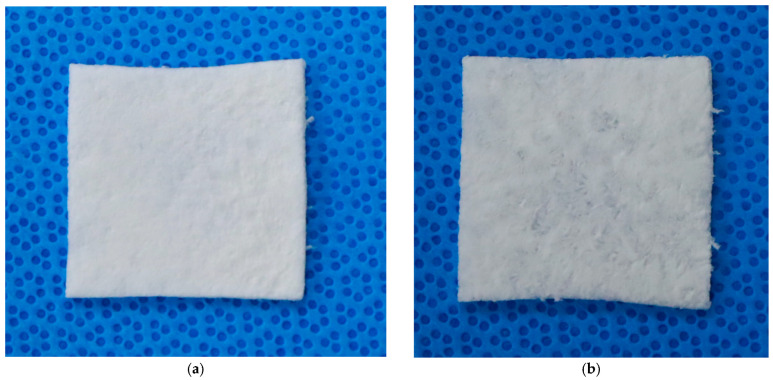
Scaffolds for full-thickness artificial skin. (**a**) Epidermal layer (**b**) Dermal layer. The denser layer was where the keratinocytes were placed, and the less dense layer was where the stromal vascular fraction (SVF) cells were placed.

The final bi-layered single scaffold included a dense collagen membrane flanked by porous layers with density gradients, mimicking the dermal–epidermal architecture. The denser layer of the two layers was where the keratinocytes were placed, and the less dense layer was where the SVF cells were placed (Figure 2).

### 2.4. Analysis of Full-Thickness Artificial Skin

The scaffolds were analyzed for collagenase degradation, tensile strength, and structural features using scanning electron microscopy (SEM). The collagenase degradation tests measured the scaffold stability over time. Scaffolds were cut into uniform pieces measuring 1 × 1 cm, and the samples were subsequently immersed in a 10 mL collagenase solution (Sigma-Aldrich) in phosphate-buffered saline (PBS) at 37 °C. At predetermined time intervals (1, 3, 5, and 7 days), the scaffolds were monitored for signs of degradation. Tensile strength testing was conducted to evaluate the mechanical properties of the scaffolds under varying preparation conditions, including gamma sterilization, using a universal testing machine (Instron). For gamma sterilization, gamma radiation was administered at a dose ranging from 25 to 40 kGy to ensure effective sterilization. Testing was also categorized into structural characterization and biocompatibility testing. The tensile strength test evaluated the mechanical properties under varying preparation conditions, while the collagenase degradation tests assessed scaffold stability. Biocompatibility was analyzed by seeding SVF cells and keratinocytes onto scaffolds and assessing cell viability and cytotoxicity over 7 days. The biocompatibility of the scaffolds was assessed by seeding SVF cells and keratinocytes onto sterilized scaffold measuring 1 × 1 cm. To evaluate cell viability and cytotoxicity, we employed the Cell Counting Kit-8 (CCK-8; Sigma-Aldrich, St. Louis, MO, USA) assay at intervals of 1, 3, 5, and 7 days post-seeding. Following the incubation period, absorbance was measured at 450 nm using a microplate reader (BioTek Instruments, Winooski, VT, USA) to quantify cell growth and proliferation, thus providing a reliable assessment of the scaffolds’ biocompatibility.

For structural characterization, SEM was utilized to examine pore size, uniformity, and structural integrity. Samples were prepared by cutting the scaffolds into small cubes of approximately 8 mm³ using a sterile scalpel and cutting board. The scaffolds were then subjected to dehydration through a graded series of ethanol baths, each lasting 15 min, to ensure the thorough removal of water from the samples. Following dehydration, the samples were critical point dried using a critical point dryer (Balzers CPD 030, Zurich, Switzerland) to replace the ethanol with CO_2_. After critical point drying, the scaffolds were coated with gold-palladium using a sputter coater (Leica EM ACE 600, Wetzlar, Germany) to a thickness of approximately 10 nm to enhance conductivity for SEM analysis. Imaging was performed with a field emission scanning electron microscope (JEOL JSM-7401F, Tokyo, Japan) operating at an accelerating voltage of 20 kV. Images were acquired in secondary electron mode to analyze the pore size, uniformity, and structural integrity of the scaffolds. The measurement process involved capturing multiple images at different magnifications to provide a comprehensive view of the scaffold architecture. Commercial acellular artificial dermis consisting of 100% collagen derived from porcine skin (Insuregraft, Atozbio, Seoul, Republic of Korea) was used as the control for comparison.

### 2.5. Cell Imaging of the Full-Thickness Artificial Skin

The cell membranes of keratinocytes and SVF cells were labeled by 2 μM PKH26 red fluorescent dye and green fluorescent dye (Sigma-Aldrich, St. Louis, MO, USA), respectively. Following labeling, the cells were cultured on a collagen scaffold to create the full-thickness artificial skin. Keratinocytes were applied to the epidermal layer, while SVF cells were applied to the dermal layer. The fabricated full-thickness artificial skin was placed on a cell culture insert (Millicell, Millipore, Burlington, MO, USA) and cultured for 5 days under standard conditions (37 °C, 5% CO_2_).

For imaging purposes, the cultured artificial skin was washed three times with PBS and then fixed in 4% paraformaldehyde for 12 h to preserve cell morphology. After fixation, the samples were washed three times with PBS, followed by dehydration and substitution processes using 70%, 95%, and 100% ethanol and xylene. The samples were then embedded in paraffin and sectioned to a thickness of 5 μm using a microtome. The sectioned samples were mounted on gelatin-coated slides and stained with hematoxylin and eosin (H&E). The overall morphology of the sample was observed using a stereomicroscope (SMZ745, Nikon, Tokyo, Japan), while cellular details were captured using an optical microscope (BX40, Olympus, Tokyo, Japan). In addition, the samples were rinsed three times with PBS, agarose-embedded, and sectioned to a thickness of 30 μm using a vibratome (Leica VT1000S, Wetzlar, Germany). The sectioned samples were subsequently mounted on glass slides, and the fluorescence expression was visualized and captured using an EVOS FL2 fluorescence microscope (Thermo Fisher Scientific, Waltham, MO, USA).

## 3. Results

### 3.1. Collagenase Degradation Test

The degradation stability of collagen-only, collagen-elastin, and acellular artificial dermis was tested under 200 U collagenase treatment. By 24 h, the commercial acellular artificial dermis was fully degraded, the collagen-only scaffolds showed gradual degradation, and the collagen-elastin scaffolds retained structural integrity. The collagen-only scaffolds were fully degraded by day 3, while the collagen-elastin scaffolds were fully degraded by day 7. Thus, collagen scaffolds might be suitable for full-thickness artificial skin scaffolds because SVF cells and keratinocytes fixed in collagen-only scaffolds would be released gradually during scaffold degradation for wound healing.

### 3.2. Tensile Strength of Scaffolds

Tensile strength tests were conducted for the acellular artificial dermis, collagen-only, and collagen-elastin scaffolds. The acellular artificial dermis showed 0.7 N in the dry tensile strength test. The collagen-only scaffolds exhibited a tensile strength of 2.2 N, while the collagen-elastin scaffolds showed a tensile strength of 4.2 N. After gamma sterilization, the collagen-only scaffolds remained stable at 2.2 N, while the collagen-elastin scaffolds exhibited a slight reduction from 4.2 N to 3.8 N. Tensile strength was also assessed under preparation conditions of primary freeze-drying, 1-ethyl-3-dimethylaminopropyl crosslinking, washing, and secondary freeze-drying. In the comparisons between the acellular artificial dermis and the collagen-only scaffold, collagen-elastin scaffolds revealed the highest tensile strength performance in all of these conditions.

### 3.3. Structural Features of the Scaffolds

**Figure 3:**
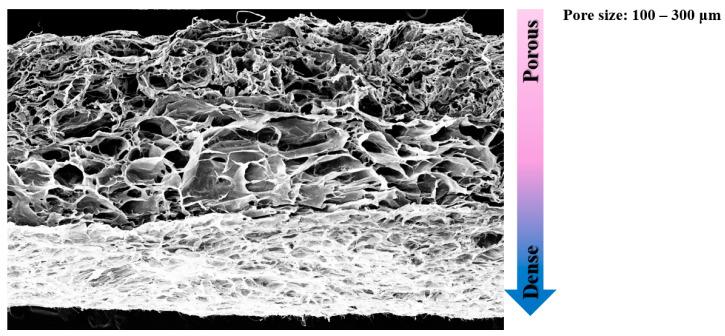
Scanning electron microscopy (SEM) image of a full-thickness artificial skin scaffold. SEM images confirmed the successful assembly of the bi-layered structure within a single scaffold.

The surface morphology and structural features of the artificial skin scaffolds were analyzed using SEM to evaluate the effects of elastin incorporation, density gradients, and bi-layer configurations. These analyses aimed to assess uniformity, pore size, and surface characteristics critical for mechanical stability and cellular integration (Figure 3).

**Figure 4:**
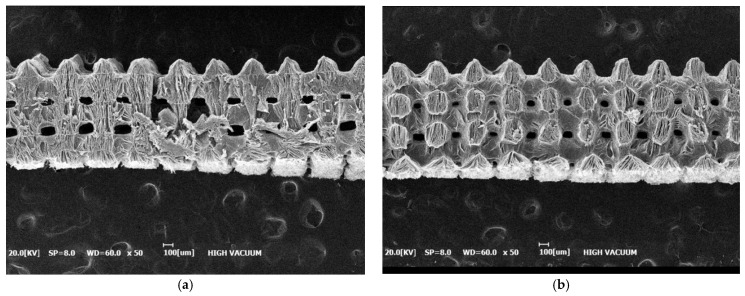
SEM images of collagen-only and collagen-elastin scaffolds. (**a**) Collagen-only scaffold, (**b**) collagen-elastin scaffold. Collagen-elastin scaffolds exhibited greater density compared to collagen-only scaffolds.

Although both collagen-only and collagen-elastin scaffolds displayed uniform pore sizes and smooth surface patterns, the SEM images revealed notable differences between the two types. The collagen-elastin scaffolds appeared to be morphologically denser than the collagen-only scaffolds, which contributed to their reduced degradability. Consequently, the collagen-elastin scaffolds were not easily degraded compared to the collagen-only scaffolds (Figure 4).

The SEM images also confirmed the successful assembly of the bi-layered structure, with two porous layers flanking the dense collagen membrane. The porous layers exhibited uniform micro-pore distribution (100–300 μm), facilitating nutrient transport and cellular infiltration. The micropore size of the scaffold into which SVF cells corresponding to the dermal layer infiltrated was set to 300 μm, and the micropore size of the scaffold into which keratinocytes corresponding to the epidermal layer infiltrated was set to 100 μm. This hierarchical structure replicates the natural dermal–epidermal interface, providing both functional and structural advantages.

**Figure 5:**
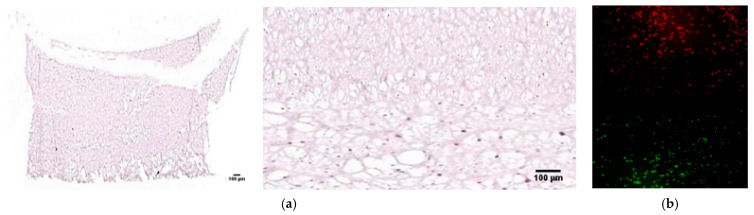
Cell image of the full-thickness artificial skin. (**a**) H&E staining (×10 and ×100). (**b**) Fluorescence imaging. These cell imaging results show red-stained keratinocytes representing the epidermal layer and green-stained SVF cells corresponding to the dermal layer.

The cell imaging results confirmed the presence of keratinocytes in the epidermal layer and SVF cells in the dermal layer, as observed through H&E staining. Additionally, fluorescence imaging revealed red-stained keratinocytes in the epidermal layer and green-stained SVF cells in the dermal layer. This configuration allowed for the separation of the two cell types within a single scaffold (Figure 5).

### 3.4. Biocompatibility

**Figure 6:**
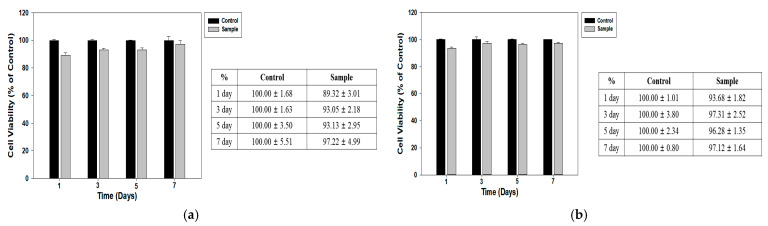
Biocompatibility test results of collagen scaffolds. (**a**) Keratinocytes. (**b**) SVF cells. The results revealed that the fabricated artificial skin scaffold was minimally toxic to both keratinocytes and SVF cells over the 7-day culture period.

Keratinocytes and SVF cells were seeded onto UV-sterilized 1 × 1 cm collagen-based artificial skin scaffolds and cultured under standard conditions (37 °C, 5% CO_2_) for 1, 3, 5, and 7 days to assess the biocompatibility of the fabricated full-thickness artificial skin constructs. Cell viability and cytotoxicity were evaluated using the CCK-8 assay, and absorbance was measured at 450 nm. The results revealed that the fabricated artificial skin scaffold was minimally toxic to both keratinocytes and SVF cells during the 7-day culture period. The cell viability, expressed as a percentage of the control group, demonstrated the consistent growth and proliferation of both cell types. Cell viability tests for keratinocytes displayed an initial viability of 89.32 ± 3.01% on day 1, which gradually increased to 97.22 ± 4.99% by day 7. Similarly, the viability of SVF cells was 93.68 ± 1.82% on day 1, which slightly improved to 97.12 ± 1.64% on day 7. These results confirm that the artificial skin scaffolds may support cellular attachment and proliferation without inducing significant cytotoxic effects (Figure 6).

## 4. Discussion

This research introduces a novel single-layer collagen scaffold featuring a gradient pore-density structure as an alternative to conventional multilayered artificial skin models. Most scaffolds designed for cell therapy products are typically single structures with a uniform pore size, making it challenging to develop two different pore sizes for two cell types within a single scaffold for full-thickness artificial skin. Additionally, the dense layer serving as the epidermal layer possesses physically stable characteristics, such as enhanced space retention and tensile strength, compared to a single-layer structure. Furthermore, this dual-layer configuration within a single scaffold may selectively guide the direction of granulation tissue formation in the dermal layer and epithelialization in the epidermal layer, facilitating effective wound healing for full-thickness skin and soft tissue defects. By utilizing variations in pore density, SVF cells and keratinocytes were effectively organized into distinct regions without the need for biochemical signaling or pre-layered construction. The results showed that the physical structure of the scaffold could significantly influence cellular spatial distribution, providing a practical and scalable solution for artificial skin applications [6,14]. This approach represents a shift from traditional tissue-engineered skin substitutes, which typically depend on pre-fabricated layers or biochemical modifications for similar cellular organization [20,21].

One of the advantages of this method is its manufacturing efficiency and suitability for clinical use. In practice, skin thickness varies across different anatomical sites, with areas such as the face requiring thinner artificial skin for effective wound coverage. The single scaffold offers this advantage as its fabrication process allows for thinner constructs compared to multilayered scaffolds, making it well suited for delicate tissue repair. Additionally, the ability to create finer single-layer scaffolds enhances the adaptability to sensitive wound environments, such as facial injuries, where flexibility and close adhesion to the skin are crucial for successful grafting. Traditional artificial skin scaffolds often necessitate multiple layers to accurately replicate the dermal–epidermal interface, which complicates the manufacturing process, extends production time, and poses handling challenges during application [22]. Multilayered scaffolds also face clinical issues where upper layers may shift or separate during application, potentially affecting integration and stability. Conversely, the single-layer scaffold developed in this study streamlines production while preserving essential cell separation. By removing the need for precise layering techniques, it has the potential to improve scalability, reproducibility, and cost-effectiveness in artificial skin fabrication [23]. The simpler structure also facilitates easier clinical application, reducing the technical skill necessary for effective wound coverage compared to complex biofabricated alternatives.

Mechanical testing demonstrated that the structural integrity of the scaffold was adequate to support cellular growth. The gradient pore density did not compromise the overall mechanical strength, ensuring stability during handling and application. Additionally, assessments of biocompatibility revealed high cell viability, suggesting that the scaffold material had no cytotoxic effects. The retention of fibroblasts and keratinocytes in their designated regions emphasizes the scaffold’s effectiveness in promoting cellular organization and potential tissue integration [24]. Notably, while the tensile strength of commercial acellular artificial dermal products is generally 0.7 N, the single-layer scaffold in this investigation exhibited a significantly higher tensile strength of 2.2 N, confirming its sufficient mechanical stability to support cell functionality and maintain structural integrity throughout the healing process.

While this study has effectively demonstrated the potential of a gradient pore-density scaffold for cell organization, several limitations must be acknowledged. First, the research was conducted in vitro, necessitating further in vivo studies to evaluate the scaffold’s effectiveness in enhancing wound healing and integration with host tissue. The interactions between the scaffold and wound environments, including inflammatory responses and vascularization, remain to be explored. The CCK-8 assay alone may not offer a comprehensive evaluation of cytotoxicity and cell proliferation. To enhance the assertions of biocompatibility, it is advisable to incorporate additional methods, such as immunostaining and flow cytometry, to assess cell apoptosis. Consequently, further in vivo studies should be undertaken to evaluate the histological and functional roles of the full-thickness artificial skin developed in this study, with particular emphasis on its efficacy in wound closure, vascularization, host integration, potential immunogenicity issues related to porcine collagen, and associated immune responses. Second, although this study confirmed the scaffold’s ability to support the adhesion of SVF cells and keratinocytes, long-term cellular behavior and tissue maturation require more investigation. Direct comparisons with conventional multilayered scaffolds are also necessary to establish the functional advantages of this strategy.

Future research should consider modifications to improve cellular integration and explore whether changes in scaffold properties, such as porosity and material composition, could enhance nutrient diffusion and cell migration in wound environments. Investigating alternative scaffold designs, including hybrid models that incorporate layered structures, may provide insights into optimizing artificial skin fabrication for a range of wound healing needs. Expanding both the mechanical and biological characterization of this scaffold, especially under dynamic conditions that simulate in vivo environments, will be critical for its clinical application. By focusing on scalable, cost-effective strategies, this study provides a fresh perspective on artificial skin scaffold design that prioritizes practical engineering solutions over mere biomimicry [6,14].

## 5. Conclusions

This research has demonstrated that a single-layer collagen scaffold with a gradient pore-density structure could facilitate the effective spatial organization of cells while offering adequate mechanical stability and biocompatibility. These findings underscore its potential as a practical and clinically relevant alternative to complex multilayered skin substitutes for wound healing purposes.

## Data Availability

The data presented in this article are not publicly available because the patient included in this study provided consent solely for academic publication in journals to contribute to medical advancements.

## Abbreviations

The following abbreviations are used in this manuscript:

H&E	Hematoxylin and Eosin
PBS	Phosphate-Buffered Saline
SVF	Stromal Vascular Fraction
SEM	Scanning Electron Microscopy
